# 2D nanoporous Ni(OH)_2_ film as an electrode material for high-performance energy storage devices†

**DOI:** 10.1039/c9ra02034a

**Published:** 2019-06-05

**Authors:** Jinjun Tian, Yan Xue, Xinping Yu, Yuanchao Pei, Hucheng Zhang, Jianji Wang

**Affiliations:** Collaborative Innovation Centre of Henan Province for Green Manufacturing of Fine Chemicals, Key Laboratory of Green Chemical Media and Reactions, Ministry of Education, School of Chemistry and Chemical Engineering, Henan Normal University Xinxiang Henan 453007 China hzhang@htu.edu.cn jwang@htu.edu.cn; School of Biological and Chemical Engineering, Nanyang Institute of Technology Nanyang Henan 473004 China

## Abstract

A two-dimensional (2D) nanoporous Ni(OH)_2_ film was successfully developed from triethanolamine (TEA) as the alkali source and soft template using a scalable hydrothermal technique. The nanostructured Ni(OH)_2_ film was flexible and translucent, and could be directly compressed on a current collector. Owing to the uniform well-defined morphology and stable structure, the Ni(OH)_2_ film binder-free electrode displayed a high specific capacity, exceptional rate capability, and admirable cycle life. The specific capacitance was 453.6 mA h g^−1^ (1633 F g^−1^) at 0.5 A g^−1^. The assembled Ni(OH)_2_//activated carbon (AC) asymmetric supercapacitor (ASC) device had an energy density of 58.7 W h kg^−1^ at a power density of 400 W kg^−1^. These prominent electrochemical properties of Ni(OH)_2_ were attributed to the high electrical conductivity, high surface area, and unique porous architecture. Free tailoring, binder-free, and direct pressing were the most significant achievements of the Ni(OH)_2_ film in the development of high-performance energy storage devices.

## Abstract 2

A two-dimensional (2D) nanoporous Ni(OH)_2_ film was successfully developed from triethanolamine (TEA) as the alkali source and soft template using a scalable hydrothermal technique.

## Graphical abstract

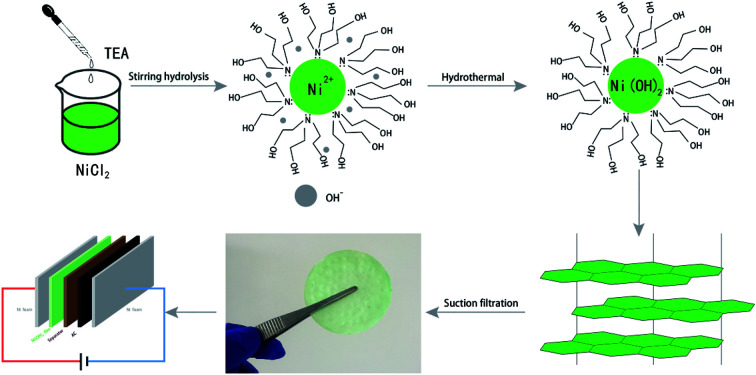

## 1. Introduction

Increasing applications of high-powered electric devices, fossil fuel depletion, and ever-increasing global environmental problems have motivated researchers to develop various high-energy density energy storage devices, mainly comprising batteries and supercapacitors.^1–6^ As one of the most promising energy storage devices, supercapacitors exhibit fast charge–discharge ability and a long cycle life, are environmentally friendly, and can provide more power density than batteries.^7–9^ However, the apparent disadvantage of electrochemical supercapacitors is their low energy density. Therefore, pseudocapacitive materials with faradaic charge storage have attracted increasing interest, such as transition metal oxides,^10,11^ hydroxides,^12,13^ and conducting polymers,^14,15^ providing fast reversible faradaic redox reactions. However, pseudocapacitance involving multiple redox reactions often suffers from low performance and a lack of cycling stability, mainly caused by the low electric conductivity and rapid decay of the electroactive surface area resulting from the instability of the microstructure/morphology upon fast and repeated charging/discharging.^16,17^ Therefore, the development of electrode materials with excellent rate capability, cycling stability, and high specific capacitance retention, remains challenging.

Among various electrode materials, transition metal hydroxides (TMH) have been investigated extensively as electrode materials owing to their abundant oxidation states that can produce reversible redox reactions.^18–20^ Ni(OH)_2_ is a promising active material for electrochemical supercapacitors owing to its high theoretical capacity (2082 F g^−1^), excellent faradaic redox reaction, abundant resources, and environmental friendliness.^21,22^ Despite these favorable factors, the capacitance performance of various Ni(OH)_2_-based materials is generally far below that of theoretical capacitors and their actual cycling life is usually poor.^23,24^ Many strategies have been devised to enhance capacitors and the actual cycling stability of fabricated Ni(OH)_2_ materials. For example, many controllable synthetic routes have been proposed for designing hierarchical nano or microstructures and framework structures to improve the contact area of electrodes and provide fast and efficient charge transfer, effectively improving electrode material utilization.^25,26^ For example, Xiong *et al.*^27^ reported that Ni(OH)_2_ nanosheets grown on nickel foam showed a specific capacitance of 2384.3 F g^−1^ at 1 A g^−1^ with a good cycling ability (∼75% of initial specific capacity remained after 3000 cycles). Liang *et al.*^28^ synthesized a Ni(OH)_2_//AC composite by depositing the soluble precursor of Ni(OH)_2_ on AC and obtained a specific capacitance of 2949 F g^−1^ at a scan rate of 20 mV s^−1^ with excellent rate and cycling performance. Furthermore, Dai *et al.*^29^ synthesized a Ni(OH)_2_ nanocrystals grown on graphene sheets as a pseudocapacitor material, which exhibited a high specific capacitance of 1335 F g^−1^ at 2.8 A g^−1^, and a high retention rate of almost 100% after 2000 cycles. Kundu *et al.*^30^ fabricated sheets with nickel metal (Ni/Ni(OH)_2_) NPs using a hydrothermal method, which achieved a specific capacitance of 450 F g^−1^ at 1 mA cm^−2^, with a retention rate of up to 90% at 15 mA cm^−2^ after 4000 cycles.

Owing to the anisotropic structure, two-dimensional nanosheets can provide shorter diffusion paths for electrons and ions, and possess significant advantages in various applications. Preparing large-area 2D nanosheets from the direct growth of nanostructures is considered an effective and straightforward method.^31,32^ The high-power electronic transport of 2D nanosheets can satisfy fast charging storage requirements. Furthermore, 2D nanomaterials can demonstrate the inherent properties of 2D structures by stacking layer-by-layer, which is conducive to the superflexibility and high mechanical properties of synchronization.^33^

Traditionally, working electrodes for energy storage devices are prepared using slurry coating technology by mixing active material, conductive carbon, and polymer binder in a certain proportion and coating on the collector.^34–36^ In fact, the binder increases the total resistance and decreases the electrode conductivity, restricts electron transport, and leads to deterioration of the electrochemical performance.^37,38^ Conductive carbon occupies a certain electrode volume and hinders the potential performance, with almost no contribution to capacitance. Therefore, constructing electrode materials with a large-area 2D structure that can be embedded directly on the current collector without any binder is worthwhile, and the expected electrode might offer a highly efficient electron-conducting channel. In this work, we rationally constructed translucent 2D Ni(OH)_2_ nanosheets with TEA as the alkali source and soft template, as electrodes for energy storage devices. The 2D Ni(OH)_2_ semitransparent film was obtained from Ni(OH)_2_ nanosheets by filtration. After cutting to a suitable size, the Ni(OH)_2_ films could be directly pressed onto Ni foam. Direct contact between active material and collector was able to transport charge efficiently. Compared with the traditional slurry coating method, the direct pressing method was simple to operate, had low equipment requirements, and made industrial batch production easy to realize. The reasonable electrode design tactics would be of great significance for high-performance energy storage devices.

## 2. Experimental section

### 2.1 Materials

Nickel chloride hexahydrate (>97%, NiCl_2_·6H_2_O), triethanolamine (TEA), potassium hydroxide (KOH), and activated carbon (AC) were purchased from Macklin Company.

### 2.2 Synthesis of 2D Ni(OH)_2_ film

Ni(OH)_2_ nanosheets were synthesized using a scalable hydrothermal technique without strict synthesis conditions and complex reaction steps. In a typical procedure, NiCl_2_·6H_2_O (2 mmol) was dissolved in H_2_O (30 mL) and TEA (12 mmol) was added slowly and stirred for 20 min. The mixture was then transferred into a 50 mL autoclave and heated at 180 °C for 12 h. After the reaction, the obtained green gels were collected by filtration and washed with distilled water. The green product was dried with filter paper at 60 °C and the Ni(OH)_2_ film was obtained by slowly removing it from the filter paper.

### 2.3 Characterization

X-ray powder diffraction (XRD, Bruker D8, Germany; Cu Kα radiation (*λ* = 0.15418 nm)) was used to investigate the phase analysis of composites from 10 to 80° (2*θ*). X-ray photoelectron spectroscopy (XPS, Kratos Amicus, Shimadzu, UK; Mg Kα radiation under 2 × 10^−6^ Pa) was employed to identify the crystal structures. Nitrogen adsorption and desorption experiments were performed using a Brunauer–Emmett–Teller measurement (BET, SSA4000). The morphologies were observed by scanning electron microscopy (SEM, ZEISS SIGMA-500, Japan) and transmission electron microscopy (TEM, JEOL JEM-2100).

### 2.4 Electrochemical measurements and calculations

The electrode was fabricated by cutting Ni(OH)_2_ film into a suitable size (1 × 1 cm) and directly pressing onto two nickel foam pieces (1 × 1 × 0.15 cm). After pressing on the tablet machine at a pressure of 10 MPa for 1 min, the working electrode was obtained and denoted as Ni(OH)_2_-direct pressing. The electrode prepared using the traditional slurry-coating method was denoted as Ni(OH)_2_-slurry-coating. In the three-electrode system, the Ni(OH)_2_ electrodes from direct pressing and slurry coating were directly used as the working electrode, Hg/HgO as the reference electrode, and a platinum plate as the auxiliary electrode. The electrochemical properties of the samples were tested by cyclic voltammetry (CV), constant current charge–discharge (GCD), and electrochemical impedance spectroscopy (EIS) at room temperature in a 6 M aqueous KOH electrolyte on an electrochemical workstation (CHI660E, Chenhua, China). EISs were recorded in the range of 10^5^–0.01 Hz with an ac perturbation of 5 mV with an open circuit voltage. The specific capacity (*C*_s_) of the as-obtained electrode was calculated according to the equation *C*_s_ = *I*Δ*t*/3.6 × *m*Δ*V*, where *C*_s_ (mA h g^−1^) is the specific capacity, and *I* (A), Δ*t* (s), *m* (g), and Δ*V* (V) are the discharge current, time, active material mass, and working potential range, respectively.

To further determine the potential of Ni(OH)_2_ film for practical applications, an optimized ASC was assembled using Ni(OH)_2_ film as the positive electrode and commercial activate carbon (AC) as the negative electrode, with a cellulose film sandwiched then immersed in 6 M KOH electrolyte. Typically, the mass ratio of negative electrode to positive electrode was set as 3.3 in the ASC device according to the charge balance equation *Q*^+^ = *Q*^−^. The active material masses were 5.3 and 1.6 mg for the negative and positive electrodes, respectively. The overall electroactive mass of the ASC was about 6.9 mg. The specific capacity (*C*_cell_), energy density (*E*), and power density (*P*) of ASC were calculated according to the following three equations, respectively:

1 *C*_cell_ = *I*Δ*t*/*MV*

2 *E* = *C*_cell_*V*/7.2

3 *P* = 3600 × *E*/Δ*t*

where *C*_cell_ (mA h g^−1^) is the specific capacity of the assembled device, *M* (*g*) is the total mass of active materials on both positive and negative electrodes, *V* (V) is the voltage window, *E* (W h kg^−1^) is the energy density, and *P* (W kg^−1^) is the power density of the ASC.

## 3. Results and discussion

### 3.1 Characterization of the product and microstructure

In the synthesis process, primary nanoparticles usually have high surface energy and are prone to agglomeration to form larger particles.^39,40^ Capping agents can inhibit the aggregation and growth of nanoparticles to a certain extent by coating nanoparticles, which controls the nanoparticle size.^41^ In general, nanomaterials can exhibit excellent optical properties owing to their smaller particle size and fewer surface defects.^42^ TEA is a weak alkali with good solubility in aqueous solution, and is low-cost and easy access. TEA acts as a capping agent to coordinate with metal ions and delay the precipitation rate, and can act as a mesopore template to produce hierarchical micro/mesoporous zeolites.^43,44^ In the present work, the Ni(OH)_2_ film was synthesized using TEA as the capping agent, template, and alkali source.

**Fig. 1:**
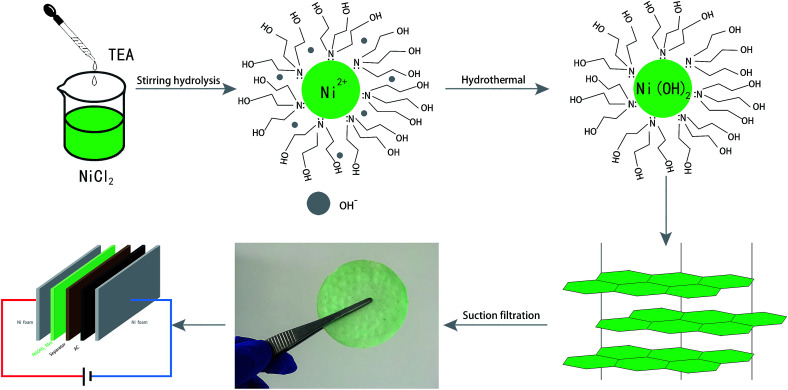
Schematic illustration of the synthesis of capped Ni(OH)_2_ nanoparticles.

The synthesis process was divided into two steps: (i) under magnetic stirring, a stable and clear green NiCl_2_ homogeneous solution was formed at room temperature, with coordination occurring between nickel ions and excess TEA (Ni^2+^ + 2(HOCH_2_CH_2_)_3_N → [Ni((HOCH_2_CH_2_)_3_N)_2_]^2+^); (ii) with the increase in reaction temperature, the coordination equilibrium was broken and the OH^−^ concentration in solution increased due to TEA electrolysis. Ni(OH)_2_ nucleation occurred *via* [Ni((HOCH_2_CH_2_)_3_N)_2_]^2+^ + 2OH^−^ → Ni(OH)_2_ + 2(HOCH_2_CH_2_)_3_N, and TEA was adsorbed around the nucleus of Ni(OH)_2_ as a capping agent. Therefore, 2D Ni(OH)_2_ nanosheets were formed by nucleation on the TEA template. The reaction mechanism showed that TEA coordinated with Ni ions to restrain spontaneous nucleation in solution, then OH^−^ attacked Ni ions to form Ni(OH)_2_ nuclei, which grew oriented by TEA template. Therefore, 2D Ni(OH)_2_ nanosheets were produced through coordination, direction, and capping by TEA molecules. The obtained green gels were collected by filtration to afford the Ni(OH)_2_ film (Fig. 1).

**Fig. 2:**
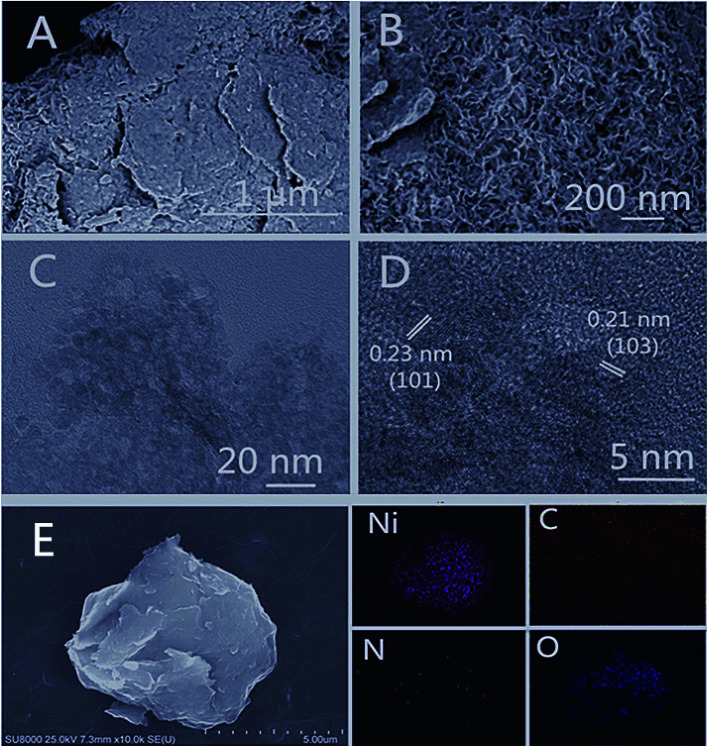
(A and B) SEM images of the as-prepared Ni(OH)_2_ film at various magnifications; (C) TEM image of Ni(OH)_2_ film; (D) high-resolution TEM image of Ni(OH)_2_ film with thickness shown in insets; (E) EDX elemental mapping images of Ni(OH)_2_ film.

The morphological and structural characteristics of the 2D Ni(OH)_2_ film were characterized by SEM and TEM. SEM images of the as-prepared Ni(OH)_2_ film, as shown in Fig. 2A and B, exhibited a uniform porous morphology. TEM micrographs showed the detailed microstructure of Ni(OH)_2_ (Fig. 2C and D). The ultrathin Ni(OH)_2_ film was observed to be interconnected and stacked evenly. The HRTEM image (Fig. 2D) showed that the Ni(OH)_2_ nanoparticles were well crystallized, with lattice spacings of 0.23 nm and 0.21 nm, corresponding to the (101) and (103) planes of Ni(OH)_2_, respectively.^45^ Energy dispersive X-ray spectroscopy (EDX) elemental mapping images (Fig. 2E) indicated that elements Ni, C, N, and O were distributed homogeneously over the nanosheets.

**Fig. 3:**
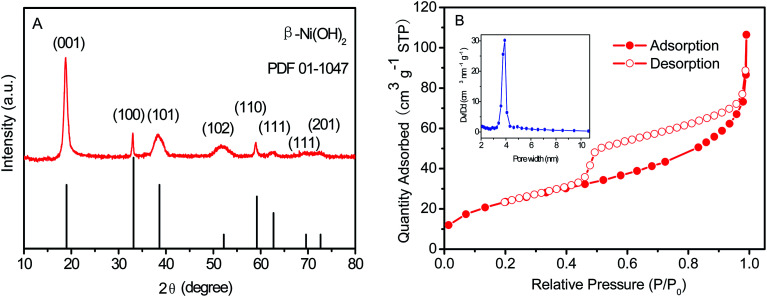
(A) XRD patterns and (B) N_2_ adsorption–desorption isotherms and BJH pore size distributions of the as-prepared Ni(OH)_2_ film.

The crystallinity and phase information of the as-prepared Ni(OH)_2_ film were investigated using XRD, with the XRD patterns shown in Fig. 3A. Obviously, all diffraction peaks were consistent with the JCPDS card (01-1047). The Ni(OH)_2_ film possessed obvious characteristic peaks at 18.9° (001), 33.3° (100), 35.59° (011), 38.6° (101), 43.6° (103), 51.8° (012), 59.4° (110), 62.5° (111), 69.5° (111), and 72.8° (201).^46^ As shown in Fig. 3B, the isotherms of Ni(OH)_2_ film displayed the characteristic mesoporous shape, which was a clear H3 hysteresis loop in a type-IV isotherm, and exhibited a large apparent specific surface area of 88.18 m^2^ g^−1^. According to the N_2_ adsorption–desorption isotherms, the pore size distribution curves of Ni(OH)_2_ film are shown in the inset of Fig. 3B. The Ni(OH)_2_ film had a mesoporous structure with a most-probable pore size of 3.58 nm, which was beneficial for high-performance supercapacitors.

**Fig. 4:**
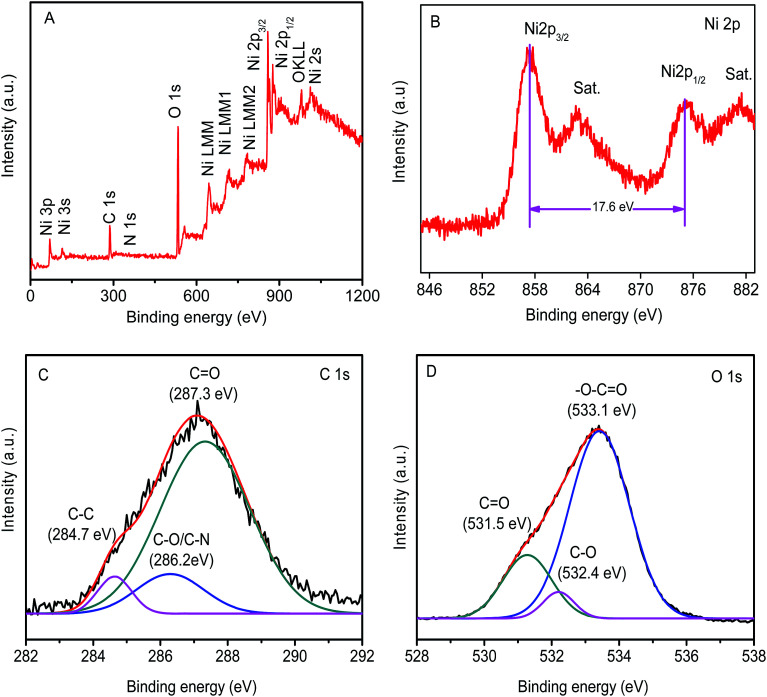
(A) XPS survey spectra, and (B) Ni2p, (C) C1s, and (D) O1s spectra of the Ni(OH)_2_ film.

The surface chemical state of the Ni(OH)_2_ film was analyzed by XPS, as shown in Fig. 4A. The survey spectra clearly indicated the occurrence of Ni, C, and O elements at their corresponding binding energies in the synthesized Ni(OH)_2_ film. The core-level spectrum of Ni 2p XPS showed two major peaks at 855.9 (Ni 2p_3/2_) and 875.7 eV (Ni 2p_1/2_), with a spin-energy separation of 17.6 eV, which was characteristic of the Ni(OH)_2_ phase (Fig. 4B).^47,48^ The high-resolution C1s spectrum (Fig. 4C) was fitted to three peaks, which were respectively assigned to C–C backbones (284.7 eV), C–N/C–O (286.2 eV), and C=O (287.3 eV), showing the decoration of N and O functionalities on the Ni(OH)_2_ film.^49^ Furthermore, the O1s spectrum was fitted into three peaks, located at 531.5, 532.4, and 533.1 eV, which were assigned to C=O, C–O, and –O–C=O (Fig. 4D).

### 3.2 Electrochemical performance of Ni(OH)_2_ film

**Fig. 5:**
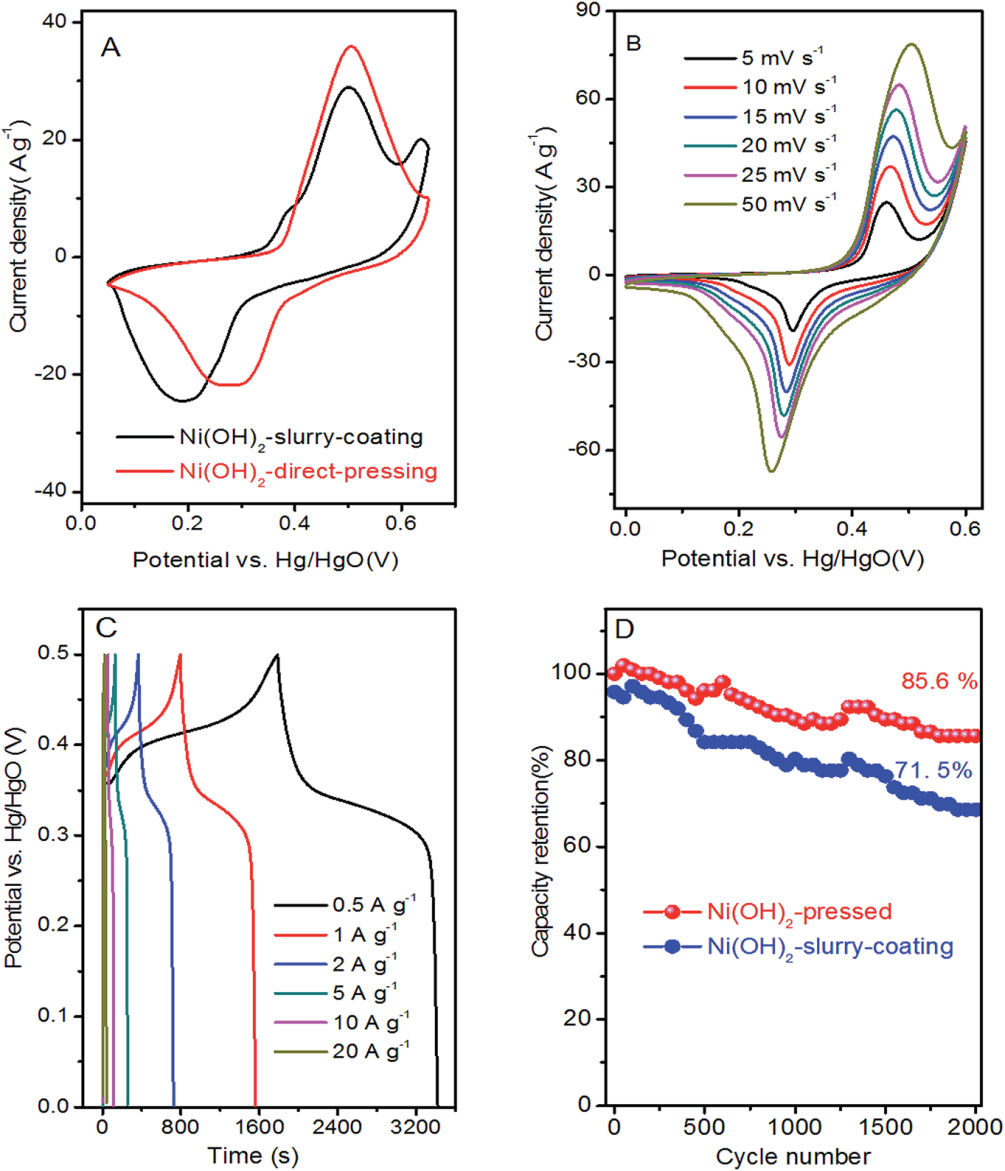
(A) CV curves of Ni(OH)_2_-direct-pressing and Ni(OH)_2_-slurry-coating electrodes at 10 mV s^−1^. (B) CV curves of Ni(OH)_2_-direct-pressing electrode at various scan rates. (C) Charge–discharge profiles at various current densities in the range of 0.5–20 A g^−1^. (D) Cycling performance of Ni(OH)_2_-direct-pressing and Ni(OH)_2_-slurry-coating electrodes at 10 A g^−1^.

The electrochemical performances of the Ni(OH)_2_ film was estimated in the electrode employing a three-electrode system in 6 M KOH aqueous solution. Fig. 5A shows a typical comparison between the Ni(OH)_2_-direct-pressing and Ni(OH)_2_-slurry-coating electrodes at 10 mV s^−1^. The CV curves showed that the Ni(OH)_2_-direct-pressing electrode had a better specific capacity than the Ni(OH)_2_-slurry-coating electrode, owing to a higher integral area. Fig. 5B shows the CV curves of the Ni(OH)_2_-direct-pressing electrode at different scan rates in the range of 5–50 mV s^−1^. The CV curves were composed of strong redox peaks, indicating that the faradaic capacitance characteristics were controlled by a faradaic redox reaction. All CV curves showed similar anodic and cathodic peaks with increasing scan rates, indicating a good reversibility at the nanostructure interface and excellent rate capability. Furthermore, as the scan rate was increased, the potential difference between the corresponding cathode peak and the anode peak increased due to polarization. The CV curves at 50 mV s^−1^ showed obvious peaks, indicating that the porous Ni(OH)_2_ film was conducive to rapid reaction. The Faraday process of Ni^2+^/Ni^3+^ accompanied by OH^−^ in the Ni(OH)_2_ film electrode material under alkaline conditions was described as follows: Ni(OH)_2_ + OH^−^ ↔ NiOOH + H_2_O + e^−^.^50^

The GCD curves, as shown in Fig. 5C, further corroborated the Faraday capacitive behavior of the Ni(OH)_2_-direct-pressing electrode. The discharge curves all showed a wide potential plateau that shortened with increasing current density. From the GCD curves, the calculated specific capacity values were 453.6, 426, 396.7, 354.2, 312.2, and 232.2 mA h g^−1^ at 0.5, 1, 2, 5, 10, and 20 A g^−1^, respectively, and the capacity retention was 51.2% under the operation current density performed 20 times. The cycling performance of the Ni(OH)_2_-direct-pressing and Ni(OH)_2_-slurry-coating electrodes at 10 A g^−1^ was measured and is shown in Fig. 5D. The cycling test suggested that the Ni(OH)_2_-direct-pressing electrode possessed a high stability for long-term applications. The initial specific capacity retention rates of the Ni(OH)_2_-slurry-coating and Ni(OH)_2_-direct-pressing electrodes were 71.5% and 85.6%, respectively, after 2000 cycles of charge and discharge. The capacity performance of the Ni(OH)_2_-direct-pressing electrode was clearly better than that of the Ni(OH)_2_-slurry-coating electrode produced using the traditional manufacture method. In the charge–discharge process, the crystal structure of the electrode active material changed, leading to deformation of the material structure. After undergoing multiple charge–discharge cycles, the active material easily detached from the current collector, resulting in capacity attenuation.^51^ Furthermore, the performance of the Ni(OH)_2_-direct-pressing electrode was similar to those of Ni(OH)_2_ and Ni-based nanostructure electrodes recently reported, as summarized in Table S1.†

**Fig. 6:**
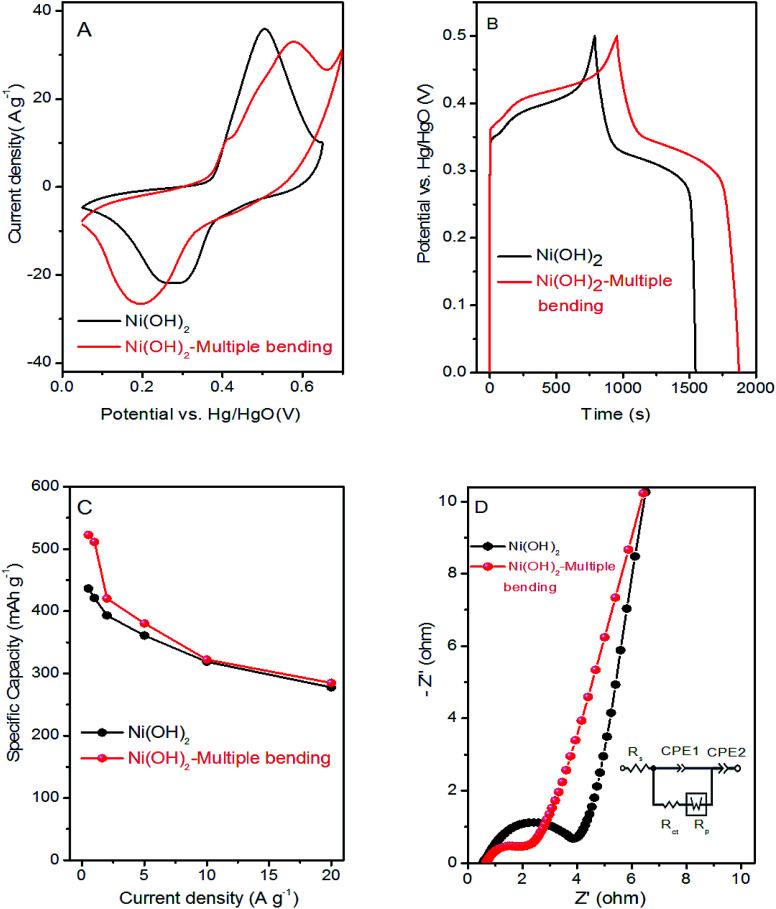
(A) CV curves at 10 mV s^−1^; (B) GCD curves at a current density of 1 A g^−1^; (C) specific capacity *vs.* current density from 0.5 to 20 A g^−1^; (D) comparison of Nyquist plots of Ni(OH)_2_ film and Ni(OH)_2_-multiple bending electrodes.

Cyclic voltammetry (CV), galvanostatic charge–discharge (GCD), and electrochemical impedance spectroscopy (EIS) of the as-formed Ni(OH)_2_ and Ni(OH)_2_-multiple bending electrodes were performed to compare their electrochemical behavior. Interestingly, the integral area of the CV curves in Fig. 6A increased after multiple bending, which indicated that the Ni(OH)_2_-multiple bending electrode possessed a much larger capacity. Fig. 6B shows that the Ni(OH)_2_-multiple bending electrode possessed better capacity performance. The calculated specific capacity of the Ni(OH)_2_-multiple bending and Ni(OH)_2_ electrodes was 421 and 511 mA h g^−1^ at a current density of 1 A g^−1^, respectively. As shown in Fig. 6C, the Ni(OH)_2_-multiple bending electrode showed superior specific capacities of 522.5, 511, 420.6, 380.6, 322.2, and 284.7 mA h g^−1^ at 0.5, 1, 2, 5, 10, and 20 A g^−1^, respectively. EIS analysis was performed to further investigate the electrochemical behavior of the Ni(OH)_2_-multiple bending electrode. Fig. 6D shows that the superior performance of the Ni(OH)_2_-multiple bending electrode could be attributed to the lower electrochemical impedance after multiple bending.

**Fig. 7:**
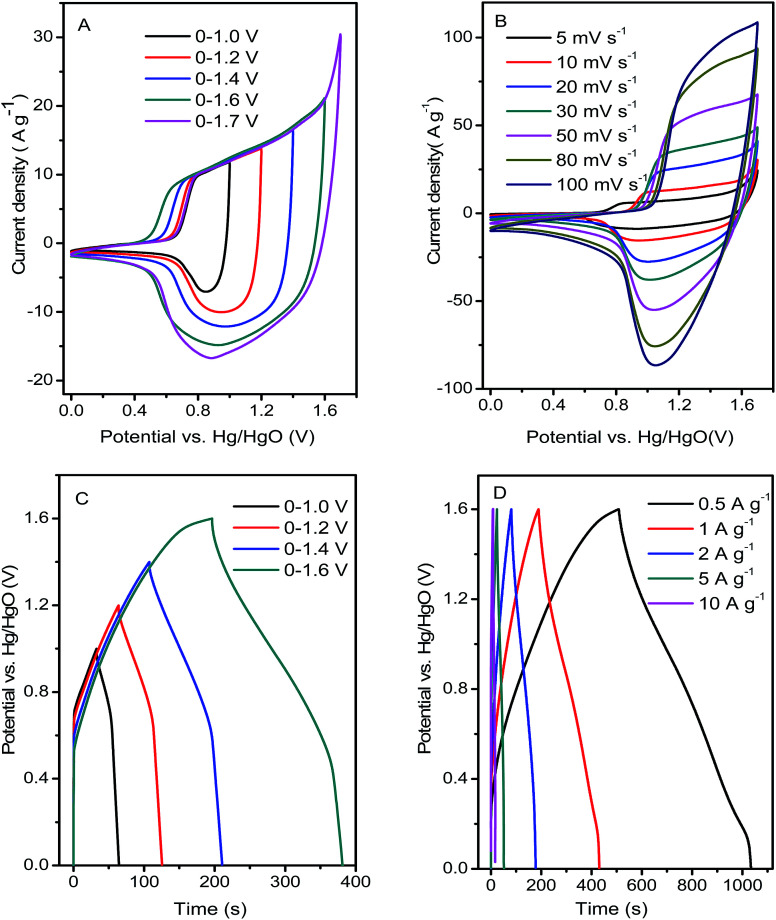
(A) CV curves within different voltage windows at 20 mV s^−1^; (B) CV curves at different scan rates; (C) GCD curves within different voltage windows at 1 A g^−1^; (D) GCD curves at different current densities.

Although Ni(OH)_2_ film attached to the Ni foam through direct pressing had a high faradaic capacity, its energy density was limited owing to the narrow voltage range. The ASC device consisting of as-synthesized Ni(OH)_2_ film and commercial AC was assembled to broaden the voltage window. The typical characteristics of the electrochemical double-layer charge storage behaviors of AC were shown by the CV curves with a rectangular shape and linear GCD curves, among others, as shown in Fig. S1.† The CVs of the ASC at different potential windows were investigated with a scan rate of 10 mV s^−1^ (Fig. 7A). The overall CV curves displayed a pair of broad and indefinite redox peaks from faradaic peaks in a two-electrode system. With expansion of the potential window from 1.0 to 1.7 V, the CV area increased significantly, and more pronounced redox reactions appeared in a wider voltage window, resulting in a higher specific capacity and energy density. Fig. 7B shows the CV curves of the ASC device at various scanning rates, in which the peak currents and CV loop areas increased with increasing scanning rate, while the peak potential varied slightly, suggesting the low overpotential resulting from the high conductivity of the two electrodes. GCD curves of different voltage windows and current densities were investigated to evaluate the charge storage capacitance of the ASC (Fig. 7C). As the voltage window was gradually increased, the discharge time increased markedly, suggesting a dramatically enhanced specific capacity in the wider voltage window. The discharge and charge curves of ASC were almost symmetrical and the inner resistance voltage drops were low at different operation current densities, reflecting good electrochemical reversibility and coulombic efficiency. The specific capacity values of the ASC device were calculated to be 45.8, 42.7, 34, 27.8, and 19.1 mA h g^−1^ at 0.5, 1, 2, 5, and 10 A g^−1^, respectively, and the capacity retention was 42% under the operation current density performed 20 times (Fig. 7D). A similar bending test was performed on ASC (Fig. S2†), resulting in acceptable electrochemical performance degradation in the contrast experiment. The specific capacity values of ASC were calculated to be 37.5, 36.2, 31.9, 25.2, and 17.9 mA h g^−1^ at 0.5, 1, 2, 5, and 10 A g^−1^, respectively, and the capacity retention was 48% under the operation current density performed 20 times (Fig. S3†), which provided a better rate capability.

**Fig. 8:**
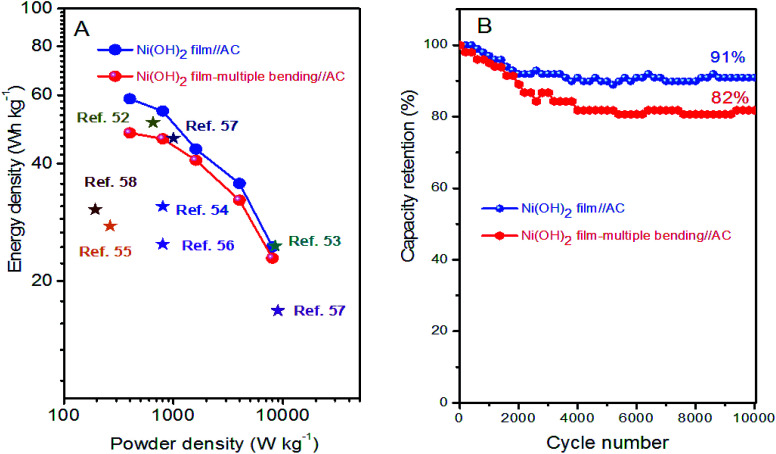
(A) Ragone plots for Ni(OH)_2_ film//AC, Ni(OH)_2_ film-multiple bending//AC and other ASCs reported previously for comparison; (B) charge–discharge cycling test at 5 A g^−1^ for Ni(OH)_2_ film//AC and Ni(OH)_2_ film-multiple bending//AC ASCs.

Energy density (*E*_cell_) and power density (*P*_cell_) are the key parameters for evaluating the ASC device. The energy and power delivery abilities of the Ni(OH)_2_ film//AC ASC were determined by Ragone plots, as shown in Fig. 8A. The *E*_cell_ of the Ni(OH)_2_ film//AC ASC reached 58.7 W h kg^−1^ at a low *P*_cell_ of 400 W kg^−1^, and decreased to 24.5 W h kg^−1^ at a *P*_cell_ of 8000 W kg^−1^. The *E*_cell_ of the Ni(OH)_2_ film-multiple bending//AC ASC decreased to 48 W h kg^−1^ at a *P*_cell_ of 400 W kg^−1^, and 22.9 W h kg^−1^ at a *P*_cell_ of 8000 W kg^−1^. The energy and power delivery abilities of the Ni(OH)_2_ film//AC and Ni(OH)_2_ film-multiple bending//AC ASCs were superior to those of previously reported supercapacitor systems, as listed in Fig. 8A. The cycling stability of the ASCs were further evaluated using 10 000 successive GCDs between 0 to 1.6 V at a current density of 5 A g^−1^. The capacity retention rates of the Ni(OH)_2_//AC and Ni(OH)_2_ film-multiple bending//AC ASCs were 91% and 82%, respectively, indicating a high charge transfer efficiency in long-term cycling. Based on the good cycling stability, the Ni(OH)_2_ film//AC ASC reported herein could serve as an efficient, long-lifetime, and high-energy-density storage system with a great potential in practical applications.

## 4. Conclusions

In summary, a facile hydrothermal method was applied to fabricate an ultrathin Ni(OH)_2_ film. Large-area 2D transparent and flexible nanostructures were formed in a straightforward manner. The Ni(OH)_2_ film was directly pressed on Ni foam as the working electrode without binder or conductive carbon. The Ni(OH)_2_ film electrode showed a high capacity of 453.6 mA h g^−1^ (1633 F g^−1^) at a current density of 0.5 A g^−1^, and a high rate capability owing to its high specific surface area and mesoporous structure. The Ni(OH)_2_-direct-pressing//AC ASC presented an outstanding specific capacitance of 45.8 mA h g^−1^ (165 F g^−1^) at 0.5 A g^−1^. The maximum energy density of the Ni(OH)_2_-film//AC ASC was 58.7 W h kg^−1^ at 400 W kg^−1^. The assembled ASC showed long-term cycling stability (91% capacity retention ratio after 10 000 cycles at 5 A g^−1^). These encouraging results confirmed that the 2D Ni(OH)_2_ film is a promising candidate for the development of high-performance energy storage devices.

## Conflicts of interest

There are no conflicts to declare.

## Supplementary Material

RA-009-C9RA02034A-s001
