# Supplementary material for: 2D nanoporous Ni(OH)2 film as an electrode material for high-performance energy storage devices
Source: RSC Adv. 2019 Jun 5;9(31):17706–16. doi: 10.1039/c9ra02034a (PMC9064570; doi:10.1039/c9ra02034a)
Supplement: RA-009-C9RA02034A-s001 [file RA-009-C9RA02034A-s001.pdf]

Electronic Supplementary Material (ESI) for RSC Advances.
This journal is © The Royal Society of Chemistry 2019

Supporting Information (SI)

Table S1. Comparison of $Ni(OH)_2$ and Ni-based electrodes recently reported in literatures

Electrodes	Capacitance	Cycling stablity	References (Year)
**$Ni(OH)_2$/NF**	2384.3 F g^{-1} at 1 A g^{-1}	3000, 75% at 5A g^{-1}	[1] (2015)
**Ni/$Ni(OH)_2$/NF**	450 F g^{-1} at 1 mA cm^{-2}	4000, 90% at 15 mA cm^{-2}	[2] (2017)
**GNS/$Ni(OH)_2$/NF**	2053 F g^{-1} at 0.3 A g^{-1}	1000, 97% at 2.5 A g^{-1}	[3] (2017)
**Ni_3S_2/NF**	1293 F g^{-1} at 5 mA cm^{-2}	1000, 69% at 25 mA cm^{-2}	[4] (2014)
**NiSe/NF**	492 F g^{-1} at 0.5 A g^{-1}	200, 91.4% at 0.5 A g^{-1}	[5] (2016)
**$Ni(OH)_2$/AC**	2949 F g^{-1} at 20 mV s^{-1}	5000, 88% at 20 mV s^{-1}	[6] (2017)
**GNS/$Ni(OH)_2$**	1335 F g^{-1} at 2.8 A g^{-1}	2000, 100% at 0.5 A g^{-1}	[7] (2010)
**$Ni(OH)_2$/NF**	340 mAh g^{-1} at 1 A g^{-1}	3000, 81.1% at 10 A g^{-1}	[8] (2018)
**$Ni(OH)_2$/NF**	453.6 mAh g^{-1} at 0.5 A g^{-1}	2000, 85.6% at 10 A g^{-1}	this work

References:

[1] X. Xiong, D. Ding, D. Chen, G. Waller, Y. Bu, Z. Wang, M. Liu, Three-dimensional ultrathin $Ni(OH)_2$ nanosheets grown on nickel foam for high-performance supercapacitors. Nano Energy, 2015， 11: 154–161.
[2] S. R. Ede, S. Anantharaj, K. T. Kumaran, S. Mishra, S. Kundu, One step synthesis of Ni/$Ni(OH)_2$ nano sheets (NSs) and their application in asymmetric supercapacitors. RSC Advances, 2017, 7(10): 5898–5911.
[3] H. Chai, X. Peng, T. Liu, X. Su, D. Jia, W. Zhou, High-performance supercapacitors based on conductive graphene combined with $Ni(OH)_2$ nanoflakes. RSC Advances, 2017, 7(58): 36617–36622.
[4] K. Krishnamoorthy, G. K. Veerasubramani, S. Radhakrishnan, S. J. Kim, One pot hydrothermal growth of hierarchical nanostructured Ni_3S_2 on Ni foam for supercapacitor application. Chemical Engineering Journal, 2014, 251: 116–122.
[5] K. Guo, F. Yang, S. Cui, W. Chen, L. Mi, Controlled synthesis of 3D hierarchical NiSe microspheres for high-performance supercapacitor design. RSC Advances, 2016, 6(52): 46523–46530.
[6] Y. Wang, X. Zhang, X. Li, X. Li, Y. Zhao, H. Wei, M. Liang, Highly dispersed ultrasmall $Ni(OH)_2$ aggregated particles on a conductive support as a supercapacitor electrode with superior performance.

Journal of Colloid and Interface Science, 2017, 490: 252–258.
[7] H. Wang, H. S. Casalongue, Y. Liang, H. Dai, $Ni(OH)_2$ Nanoplates Grown on Graphene as Advanced Electrochemical Pseudocapacitor Materials. Journal of the American Chemical Society, 2010, 132(21): 7472–7477.
[8] J. Guo, Y. Zhao, N. Jiang, A. Liu, L. Gao, Y. Li, T. Ma, In-Situ Grown $Ni(OH)_2$ Nanosheets on Ni Foam for Hybrid Supercapacitors with High Electrochemical Performance. Journal of The Electrochemical Society, 2018, 165(5), A882–A890.

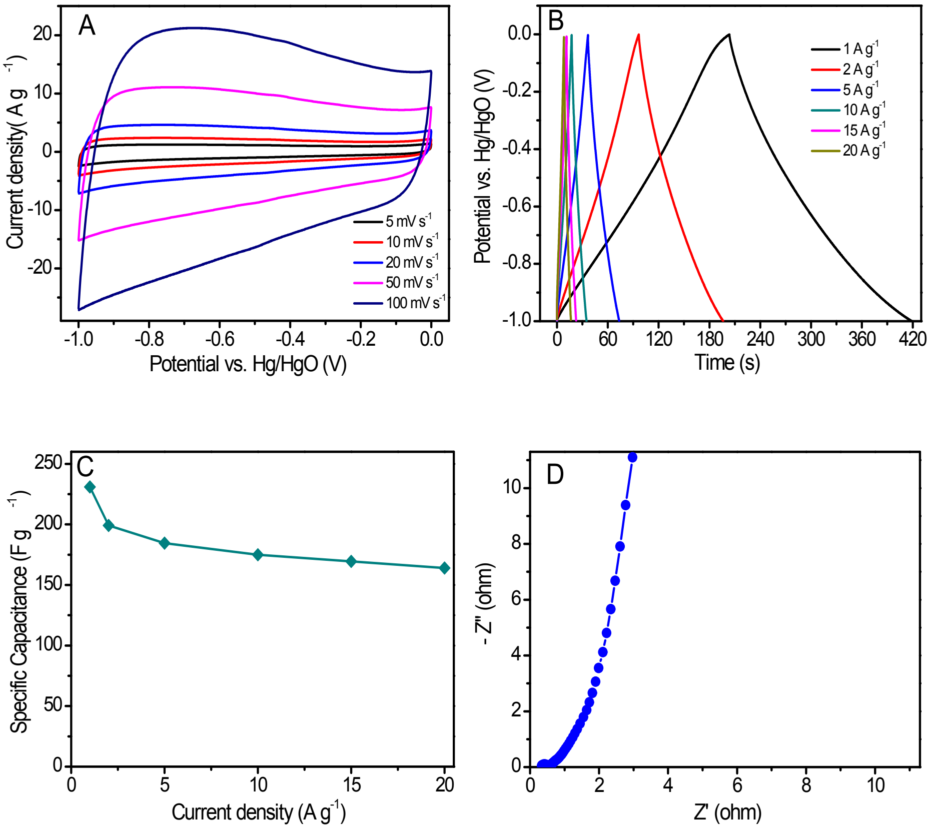

Fig. S1 (A) CV curves at different scan rates, (B) GCD curves under different discharging current densities, (C) Specific capacitance at different current densities, (D) Nyquist impedance plots of activated carbon's in 6 M KOH aqueous solution and the activated carbon mass loading is about 5 mg.

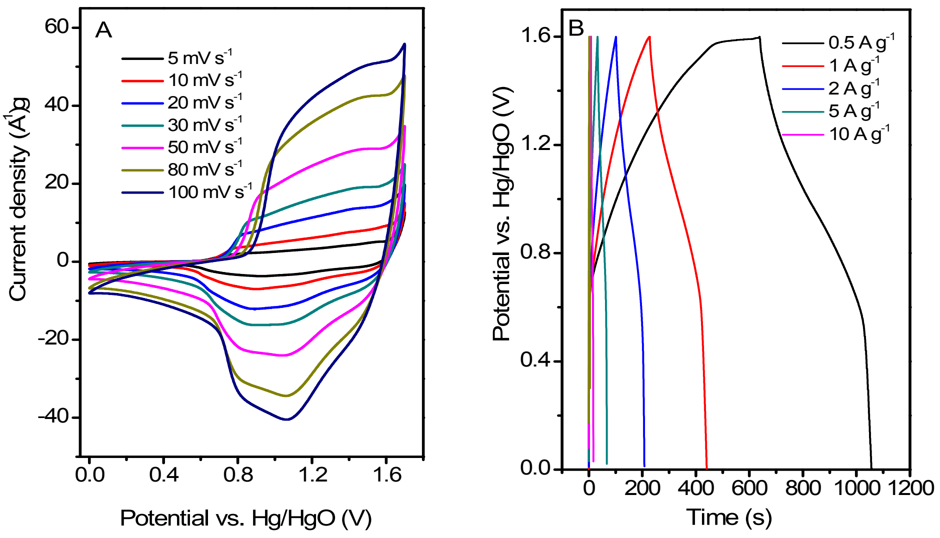

Fig. S2 (A) CV curves at various scan rates, (B) GCD curves at different current density of $Ni(OH)_2$ film-multiple bending//AC ASC in 6 M KOH.

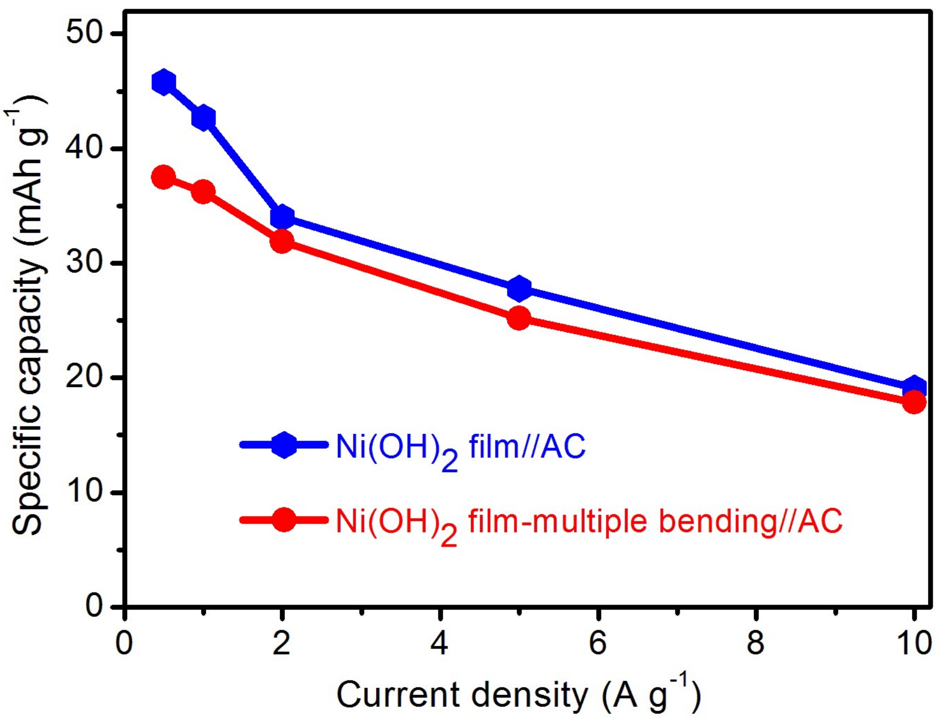

Fig. S3 the specific capacity of $Ni(OH)_2$ film//AC and $Ni(OH)_2$ film-multiple bending//AC ASCs at different current densities.
